# Comparative change in HbA1c and weight outcomes between obese and non-obese adults with type 2 diabetes mellitus treated with SGLT-2 inhibitors or GLP-1 receptor agonists

**DOI:** 10.3389/fendo.2026.1847755

**Published:** 2026-08-31

**Authors:** Omar A. Almohammed, Omar A. Alshaya, Lama A. Alduhayshi, Sara T. Alshaibani, Meshal S. Alhamed, Mohammed A. Alzeer, Othman M. Alabdullah, Mohamed F. Ibn Saqyan, Majed S. Al Yami, Nader Bin Sheraim, Sumaya N. Almohareb, Osamah M. Alfayez, Ghazwa B. Korayem

**Affiliations:** 1 Department of Clinical Pharmacy, College of Pharmacy, King Saud University, Riyadh, Saudi Arabia; 2 Pharmacoeconomics Research Unit, College of Pharmacy, King Saud University, Riyadh, Saudi Arabia; 3 Department of Pharmacy Practice, College of Pharmacy, King Saud bin Abdulaziz University for Health Sciences, Riyadh, Saudi Arabia; 4 King Abdullah International Medical Research Center, Riyadh, Saudi Arabia; 5 Pharmaceutical Care Department, Ministry of National Guard-Health Affairs, Riyadh, Saudi Arabia; 6 College of Medicine, King Saud University, Riyadh, Saudi Arabia; 7 Pharmaceutical Care Services, King Abdullah Bin Abdulaziz University Hospital, Riyadh, Saudi Arabia; 8 Department of Pharmacy Practice, College of Pharmacy, Qassim University, Qassim, Buraydah, Saudi Arabia; 9 Department of Pharmacy Practice, College of Pharmacy, Princess Nourah Bint Abdulrahman University, Riyadh, Saudi Arabia

**Keywords:** body mass index, GLP-1 receptor agonists, HbA1c, obesity, SGLT-2 inhibitors, type 2 diabetes mellitus, weight loss

## Abstract

**Background:**

Obesity and type 2 diabetes mellitus (T2DM) are closely linked through shared pathophysiological mechanisms affecting glucose metabolism and insulin resistance. Sodium–glucose cotransporter-2 inhibitors (SGLT2i) and glucagon-like peptide-1 receptor agonists (GLP-1RA) are widely recommended for patients with T2DM who are overweight or obese because of their favorable effects. However, whether baseline obesity influences the glycemic response to these agents in real-world clinical practice remains unclear.

**Methods:**

This multicenter retrospective study included adults with T2DM initiated on either SGLT2i or GLP-1RA at three healthcare centers in Riyadh, Saudi Arabia. Patients were stratified into obese (BMI ≥30 kg/m²) and non-obese (BMI <30 kg/m²). Changes in HbA1c, body weight, and BMI were evaluated at 3, 6, 9, and 12 months. Between-group comparisons were performed using independent t-tests, followed by mixed-effects models for repeated measures (MMRM) to adjust for potential confounders. Secondary outcomes included the proportions of patients achieving ≥5% weight loss, HbA1c <7.0% (among those uncontrolled at baseline), and HbA1c reductions of ≥0.5% and ≥1.0%.

**Results:**

A total of 1,016 patients were included, of whom 73.4% were obese. Obese patients were associated with greater reductions in absolute body weight and BMI at 3, 6, 9, and 12 months than non-obese patients, although their higher baseline body weight may have contributed to these differences. Responder analysis showed that obesity was associated with a higher likelihood of achieving ≥5% weight loss at months 3 (21.9% vs. 12.0%, p=0.0016) and 6 (27.2% vs. 15.5%, p=0.0086). In the adjusted MMRM, obesity was associated with greater least-squares mean reductions in weight and BMI (p<0.05), whereas HbA1c reductions and responder rates (HbA1c <7.0%, ≥0.5%, and ≥1.0% reductions) were statistically comparable between groups.

**Conclusion:**

These findings suggest that baseline obesity was associated with greater reductions in anthropometric measures following SGLT2i or GLP-1RA therapy after multivariable adjustment, whereas improvements in glycemic control were similar between obese and non-obese patients. As these findings are hypothesis-generating, prospective studies are needed to determine whether baseline BMI modifies treatment response.

## 1 Introduction

The risk of type 2 diabetes mellitus (T2DM) increases progressively as body mass index (BMI) rises. This has been attributed to increased adiposity, which contributes to the development of T2DM through complex pathophysiological mechanisms, including impaired β-cell function, altered adipose tissue signaling, and multi-organ insulin resistance. Importantly, these metabolic abnormalities may improve or normalize with sufficient weight loss, highlighting the therapeutic importance of weight management in diabetes care (1).

Sodium–glucose cotransporter-2 inhibitors (SGLT-2i) are antihyperglycemic agents that lower plasma glucose by inhibiting SGLT-2 proteins in the proximal renal tubules, thereby reducing glucose reabsorption and increasing urinary glucose excretion. Agents such as canagliflozin, dapagliflozin, and empagliflozin reduce the renal threshold for glucose and decrease glucose reabsorption by approximately 40%-50%, resulting in an average HbA1c reduction of 0.6%-0.9% in patients with T2DM (2, 3). In addition to improving glycemic control, SGLT-2i are associated with modest but consistent weight loss of about 1.5–2 kg and reductions in blood pressure, effects that are particularly beneficial in overweight and obese patients (4). Furthermore, large clinical trials have demonstrated their cardiovascular and renal protective effects, leading to their inclusion in major international diabetes treatment guidelines (5, 6).

Another widely used approach for the management of T2DM and obesity is treatment with glucagon-like peptide-1 receptor agonists (GLP-1RA), which are incretin-based therapies (5–7). According to the American Diabetes Association guidelines, GLP-1RA are recommended for patients who do not achieve glycemic targets with metformin, as well as for those with established cardiovascular or renal disease or marked hyperglycemia (5). These agents improve glycemic control by enhancing glucose-dependent insulin secretion, suppressing inappropriate glucagon release, and delaying gastric emptying (5–7). In a meta-analysis of 31 randomized controlled trials (RCTs), GLP-1RA reduced HbA1c by a pooled mean difference of −0.78% (95% CI, −0.97% to −0.60%) (7). Beyond glucose lowering, GLP-1RA induce clinically meaningful weight loss through appetite suppression and increased satiety, which has led to regulatory approval of selected agents for chronic weight management in individuals with obesity and weight-related comorbidities (8, 9). The pooled effect of GLP-1RA on body weight demonstrated a mean difference of -4.05 kg (95% CI, -5.02 to -3.09 kg) (7).

Both GLP-1RA and SGLT-2i are contemporary glucose-lowering therapies that improve glycemic control, promote weight loss, and reduce cardiovascular risk through distinct mechanisms of action. GLP-1RA primarily reduce caloric intake via central appetite regulation, whereas SGLT-2i promote weight loss through increased urinary glucose excretion (9, 10). Current guidelines recommend both drug classes for patients with T2DM who are overweight or obese, and selected GLP-1RA have also received approval for weight management in individuals without diabetes (9).

Overall, the available evidence suggests that both GLP-1RA and SGLT-2i are effective in reducing HbA1c levels in both obese and non-obese T2DM patients. While some studies indicate that obese patients may experience weight loss and potentially more significant HbA1c reductions with these treatments, other findings suggest that the HbA1c lowering efficacy is largely independent of baseline BMI (11, 12). The collective findings support the use of these medications for managing T2DM across different BMI categories, with individual patient characteristics influencing the magnitude of response (13). Large, randomized trials evaluating semaglutide, liraglutide, and empagliflozin consistently demonstrated clinically meaningful weight reduction, particularly in patients with higher baseline BMI (14, 15). These findings are further supported by comparative meta-analyses showing that both GLP-1RA and SGLT-2i produce significant weight loss across overweight and obese populations (9).

Important gaps remain regarding whether baseline BMI modifies treatment response in routine clinical practice. Clinical trial populations are generally highly selected and may not fully represent the heterogeneity encountered in real-world settings, where patients often have multiple comorbidities, varying degrees of medication adherence, concomitant therapies, and diverse prescribing patterns. Therefore, this study aims to evaluate whether baseline BMI influences changes in HbA1c and body weight among adults with T2DM treated with either an SGLT-2i or a GLP-1RA in real-world practice, after accounting for drug class and baseline clinical differences.

## 2 Methods

### 2.1 Study design, setting and patient selection

This multicenter retrospective, population-based observational study was conducted at three major healthcare institutions in Riyadh, Saudi Arabia (SA); King Saud University Medical City (KSUMC), King Abdulaziz Medical City–Ministry of National Guard (KAMC), and King Abdullah bin Abdulaziz University Hospital (KAAUH). The study included patients treated between January 2015 and May 2025. Institutional review boards at all participating centers approved the study protocol (Ref. Nos. E-20-4950; SP22R/241/09; and 21/0938/IRB, respectively). Given the retrospective design, the requirement for informed consent was waived.

Eligible participants were adults (≥ 18 years) with a confirmed diagnosis of T2DM who received care at one of these centers and were initiated on SGLT-2i or GLP-1RA during the study period, but not both therapies. Lifestyle interventions such as diet and physical activity were not captured due to the retrospective nature of the study. Patients were stratified by their baseline BMI into obese (BMI ≥ 30 kg/m²) and non-obese (BMI < 30 kg/m²) groups. Exclusion criteria included patients with other types of diabetes (e.g., type 1 diabetes or gestational diabetes), insufficient follow-up, or missing key baseline clinical or laboratory data.

### 2.2 Data collection and variables

Data were retrospectively extracted from electronic medical records at KSUMC, KAMC, and KAAUH and entered into the Research Electronic Data Capture (REDCap^®^) platform. All data entries were reviewed to ensure completeness and accuracy. Baseline variables included demographic characteristics (e.g., age, sex, height, weight, and BMI), duration of T2DM, smoking status, and comorbidities (e.g., hypertension, dyslipidemia, chronic kidney disease (CKD), and cardiovascular diseases). Baseline and follow-up laboratory parameters included HbA1c and fasting blood glucose (FBG). Medication history captured before initiating SGLT-2i or GLP-1RA therapies and information on the prescribed SGLT-2i or GLP-1RA, including agent name. Follow-up data were obtained from routine follow-up clinic visits within 12 months of treatment initiation and included weight, BMI, blood pressure, HbA1c, FBG, and renal function tests.

### 2.3 Outcome measures

The primary outcomes of the study were the absolute changes in HbA1c, body weight (kg), and body mass index (BMI), as well as the relative percentage weight change (%) calculated from baseline to each follow-up time points after initiating SGLT-2i or a GLP-1RA, after accounting for the effect of drug classes and baseline clinical differences. To account for variability in visit schedules, follow-up visits were mapped to specific time windows based on the number of days from the treatment initiation date. The windows were defined as follows: 3-month (14 to <135 days), 6-month (135 to <225 days), 9-month (225 to <315 days), and 12-month (315 to <405 days). In cases where a patient had multiple recorded visits within a single time window, the visit closest to the target date (90, 180, 270, or 360 days, respectively) was selected to represent that time point.

### 2.4 Statistical analysis

Descriptive statistics were used to summarize patient characteristics and study outcomes. Continuous variables were reported as means (± standard deviation, SD), whereas categorical variables were presented as frequencies and percentages. Between-group comparisons for continuous variables were performed using independent-samples t-tests. Where variances were unequal (folded F test P < 0.05), the Satterthwaite approximation was utilized for the mean difference (MD), 95% confidence intervals (95% CI), and P-value calculations. A P-value <0.05 was considered statistically significant.

To provide additional clinical context beyond mean continuous changes, we evaluated the proportion of patients achieving specific clinical targets at 3, 6, 9, and 12 months: ≥5% body weight reduction, an absolute HbA1c reduction of ≥0.5% and ≥1.0%, and HbA1c level <7.0%. Notably, the achievement of HbA1c <7.0% was analyzed strictly among the subset of patients presenting with an uncontrolled baseline HbA1c ≥7.0%). To properly account for follow-up attrition, these proportions were calculated using the exact denominator of patients with available data at each respective time point. The proportions of patients achieving these targets were compared between the obese (BMI ≥30 kg/m²) and non-obese (BMI <30 kg/m²) cohorts using Chi-square tests, with results reported as discrete counts and percentages (n/N [%]).

To account for repeated assessments and missing data over time, longitudinal changes in absolute body weight, percentage weight change (%), BMI, HbA1c, and fasting blood glucose (FBG) over 12 months were analyzed using Mixed-Effects Model for Repeated Measures (MMRM) under the missing-at-random (MAR) assumption. The model included the change (or percentage change) from baseline at each visit as the dependent variable. Fixed categorical effects included baseline BMI cohort (obese vs. non-obese; modeled strictly as a categorical grouping variable), time points (3, 6, 9, and 12 months), and the BMI-by-time interaction. To ensure robust adjustment for confounding, the models were adjusted for continuous baseline covariates including age, HbA1c, baseline body weight, FBG, eGFR, SBP, and DBP. Furthermore, the models were adjusted for gender, smoking status, and a comprehensive suite of clinical comorbidities: hypertension, dyslipidemia, hypothyroidism, hyperthyroidism, cardiovascular diseases (mainly ASCVD), heart failure, chronic kidney disease, retinopathy, neuropathy, and history of amputations. Baseline pharmacotherapy was also meticulously adjusted for by including specific drug classes as independent binary covariates: GLP-1RA, SGLT-2i, metformin, sulfonylureas, dipeptidyl peptidase-4 (DPP-4) inhibitors, thiazolidinediones, alpha-glucosidase inhibitors, meglitinides, insulin (any formulation), antihypertensive agents, dyslipidemia medications, antiplatelet therapy, and digoxin. To prevent perfect multicollinearity and model over-parameterization, adjustment was maintained at the drug-class level rather than by individual agents. To determine the most appropriate covariance structure for within-subject correlations, various matrices were empirically tested. The final structures were selected based on achieving optimal model convergence and the lowest Akaike Information Criterion (AIC) values; specifically, an Autoregressive [AR(1)] structure provided the best fit for glycemic outcomes (HbA1c and FBG), while a Compound Symmetry (CS) structure was optimal for body weight and BMI. Across all models, degrees of freedom were calculated via the Kenward-Roger approximation. Prior to finalizing the models, standard residual diagnostics were performed to confirm the assumptions of normality and homoscedasticity. Results are presented as least squares (LS) mean changes from baseline (± standard error, SE), with between-group differences reported as mean differences and corresponding 95% CI. Furthermore, subgroup analyses examined differences between SGLT-2i and GLP-1RA agents by comparing changes in HbA1c, body weight, and BMI across the treatment groups. All data management and statistical analyses were performed using SAS software Version 9.4 (SAS Institute Inc., Cary, NC, USA).

To further account for potential confounding by indication—specifically the unequal clinical allocation of pharmacological therapies between the obese and non-obese cohorts—all relevant baseline antidiabetic medications (including GLP-1 RA, SGLT-2i, and other background therapies) were included as fixed covariates in the MMRM analysis. To maintain clinical transparency and illustrate the impact of this multivariable adjustment, both unadjusted raw clinical trajectories and the fully adjusted MMRM marginal means are presented. The MMRM framework was specifically selected to robustly handle follow-up attrition; by utilizing a likelihood-based approach under the MAR assumption, the model natively incorporates all available partial longitudinal data, preventing the bias and loss of statistical power associated with complete-case analysis. Propensity score matching (PSM) was deliberately not utilized; because baseline obesity strongly dictates the clinical prescription of GLP-1 RA versus SGLT-2i, matching would violate the positivity assumption, resulting in severe cohort depletion and the loss of real-world external validity.

## 3 Results

### 3.1 Baseline characteristics

**Figure 1:**
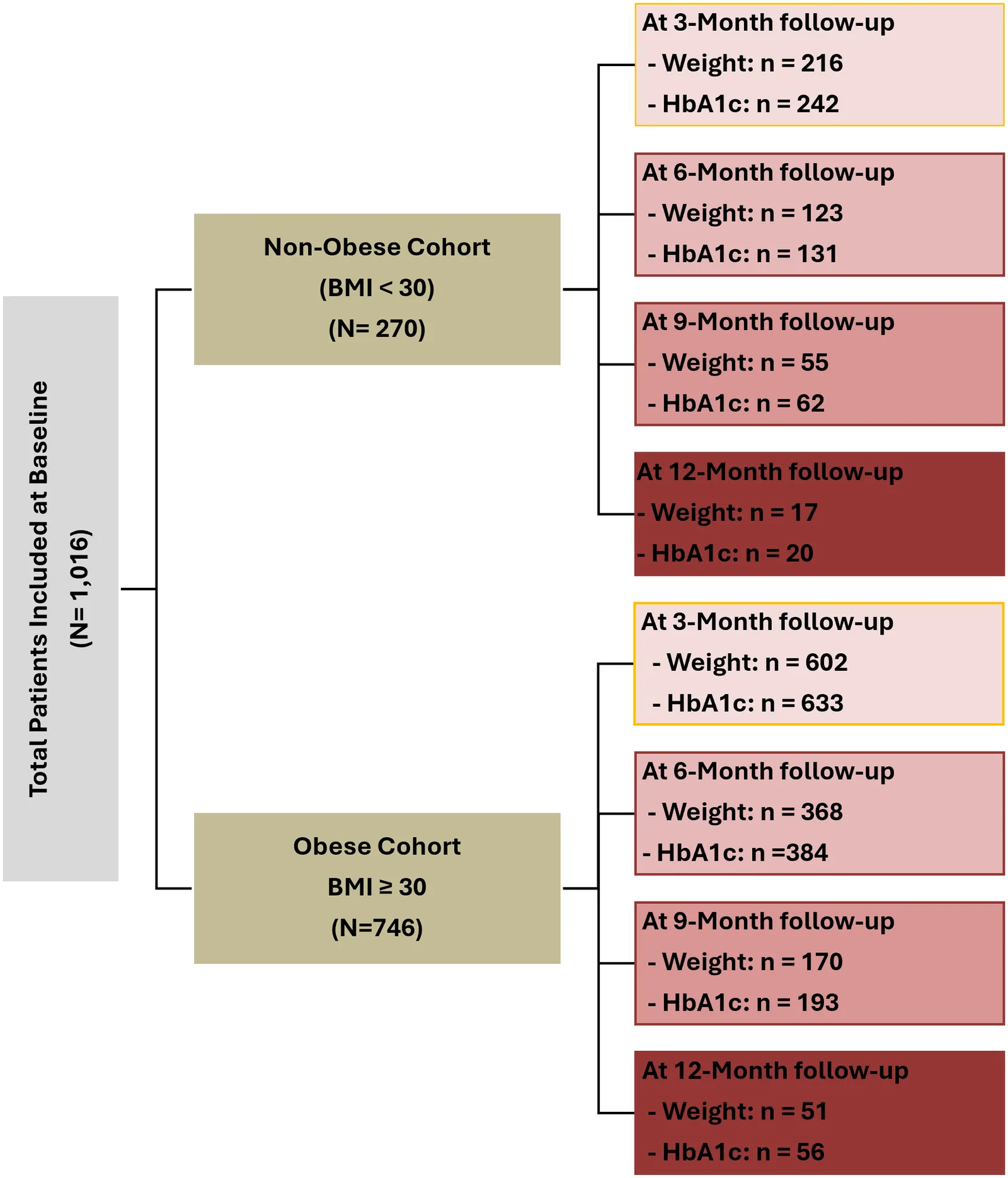
Flow diagram of included patients and available follow-up data. This flowchart depicts the availability of primary clinical data (Weight and HbA1c) at each follow-up interval for the obese (BMI ≥30) and non-obese (BMI <30) cohorts. Due to the retrospective, real- world nature of the observational data, the number of patients with available readings varies slightly depending on the specific outcome measured at a given clinic visit (e.g., a patient may have completed laboratory draws for HbA1c without a corresponding anthropometric weight).

A total of 1,016 patients with T2DM who were initiated on SGLT-2i or GLP-1RA were included in the study. Of these, 270 patients (26.6%) had a BMI < 30 kg/m² and 746 (73.4%) were classified as obese (BMI ≥ 30 kg/m²) (**Figure 1**). The overall mean age was 57.93 ± 11.3 years, with patients in the non-obese group being slightly older than those in the obese group (59.96 ± 11.0 vs. 57.20 ± 11.3 years, respectively). Females constituted the majority of the cohort (58.1%), with a higher proportion classified as obese (479/590; 81.2%) compared to males (267/426; 62.7%).

Among those with a documented history of T2DM diagnosis (~60%), most had long-standing diabetes, with 19.2% having T2DM for more than 20 years. Hypertension (71.3%) and dyslipidemia (75.1%) were highly prevalent across both BMI groups. Cardiovascular diseases, mainly atherosclerotic cardiovascular diseases (ASCVD) were documented in 17.1% of patients, and CKD in 7.5%. Detailed baseline demographic and clinical characteristics are presented in **Table 1**.

**Table 1:** Patients baseline characteristics.

Characteristic	Overall	BMI group	
		BMI < 30	BMI ≥ 30
No of patients	1016 (100.0)	270 (26.6)	746 (73.4)
Age, years	57.93 ± 11.3	59.96 ± 11.0	57.20 ± 11.3
Age > 55 years	643 (63.3)	193 (71.5)	450 (60.3)
Gender			
Female	590 (58.1)	111 (41.1)	479 (64.2)
Male	426 (41.9)	159 (58.9)	267 (35.8)
Smoking status			
Never smoked	526 (51.8)	111 (41.1)	415 (55.6)
Current smoker	78 (7.7)	34 (12.6)	44 (5.9)
Former smoker	37 (3.6)	8 (3.0)	29 (3.9)
Undocumented	375 (36.9)	117 (43.3)	258 (34.6)
Family history of T2DM	134 (13.2)	22 (8.2)	112 (15.0)
Diagnosed with T2DM within			
< 1 year	14 (1.4)	1 (0.4)	13 (1.7)
1–5 years	77 (7.6)	13 (4.8)	64 (8.6)
6–10 years	133 (13.1)	37 (13.7)	96 (12.9)
11–15 years	103 (10.1)	26 (9.6)	77 (10.3)
16–20 years	92 (9.1)	24 (8.9)	68 (9.1)
> 20 years	195 (19.2)	36 (13.3)	159 (21.3)
Undocumented	402 (39.6)	133 (49.3)	269 (36.1)
Comorbidities and diabetes complications			
Hypertension	724 (71.3)	186 (68.9)	538 (72.1)
Dyslipidemia	763 (75.1)	222 (82.2)	541 (72.5)
Hypothyroidism	160 (15.8)	33 (12.2)	127 (17.0)
Hyperthyroidism	11 (1.1)	4 (1.5)	7 (0.9)
Cardiovascular diseases, mainly ASCVD	174 (17.1)	61 (22.6)	113 (15.2)
Myocardial infarction	125 (12.3)	45 (16.7)	80 (10.7)
Stenosis^*^	75 (7.4)	22 (8.2)	53 (7.1)
Left ventricular hypertrophy	23 (2.3)	8 (3.0)	15 (2.0)
Stable angina	24 (2.4)	10 (3.7)	14 (1.9)
Unstable angina	21 (2.1)	4 (1.5)	17 (2.3)
Peripheral arterial disease claudication	5 (0.5)	0 (0.0)	5 (0.7)
Stroke	39 (3.8)	11 (4.1)	28 (3.8)
Transient ischemic attack	19 (1.9)	7 (2.6)	12 (1.6)
Heart failure	86 (8.5)	23 (8.5)	63 (8.5)
HFrEF (Ejection fraction < 40%)	19 (1.9)	8 (3.0)	11 (1.5)
HFmrEF (Ejection fraction 41-49%)	10 (1.0)	3 (1.1)	7 (0.9)
HFpEF (Ejection fraction >= 50%)	46 (4.5)	9 (3.3)	37 (5.0)
Not documented	11 (1.1)	3 (1.1)	8 (1.1)
Chronic Kidney Disease	76 (7.5)	25 (9.3)	51 (6.8)
Stage 1	1 (0.1)	0 (0.0)	1 (0.1)
Stage 2	7 (0.7)	4 (1.5)	3 (0.4)
Stage 3	32 (3.2)	10 (3.7)	22 (3.0)
Stage 4	10 (1.0)	3 (1.1)	7 (0.9)
Stage 5	2 (0.2)	0 (0.0)	2 (0.3)
Not documented	24 (2.4)	8 (3.0)	16 (2.1)
Retinopathy	89 (8.8)	21 (7.8)	68 (9.1)
Neuropathy	50 (4.9)	18 (6.7)	32 (4.3)
Amputations	6 (0.6)	1 (0.4)	5 (0.7)

Numbers are presented as frequency (%) or mean ± SD.

* Stenosis: carotid, coronary or lower extremity stenosis > 50%;

BMI, Body Mass Index; T2DM, Type 2 Diabetes Mellitus; ASCVD, Atherosclerotic Cardiovascular Disease.

### 3.2 Baseline medications and diabetes therapy

The majority of patients were receiving background antihypertensive medications (70.1%), lipid-lowering medications (83.3%, with statin use), and antiplatelet therapies (43.9%, with aspirin use). Regarding antihyperglycemic therapy, 89.6% were on non-insulin agents, and 54.5% were receiving insulin therapy. Metformin was used in 82.0% of patients, with a comparable proportion across groups. GLP-1RA (liraglutide or semaglutide) were more frequently used in obese patients (67.8%), whereas SGLT-2i (mainly dapagliflozin) were more common in the non-obese group (65.2%). Medication distribution is summarized in **Table 2**.

**Table 2:** Patients’ medications.

Characteristic	Overall	BMI group	
		BMI < 30	BMI ≥ 30
No of patients	1016 (100.0)	270 (26.6)	746 (73.4)
Hypertension medications^*^	712 (70.1)	188 (69.6)	524 (70.2)
ACEIs	255 (25.1)	66 (24.4)	189 (25.3)
ARBs	365 (35.9)	92 (34.1)	273 (36.6)
Beta-blockers	301 (29.6)	89 (33.0)	212 (28.4)
CCBs	300 (29.5)	77 (28.5)	223 (29.9)
Thiazide diuretics	110 (10.8)	19 (7.0)	91 (12.2)
Loop diuretics	106 (10.4)	16 (5.9)	90 (12.1)
Aldosterone antagonists	16 (1.6)	4 (1.5)	12 (1.6)
Sacubitril/valsartan	10 (1.0)	5 (1.9)	5 (0.7)
Dyslipidemia medications*			
Statin	846 (83.3)	235 (87.0)	611 (81.9)
Ezetimibe	79 (7.8)	23 (8.5)	56 (7.5)
PCSK9 inhibitors	3 (0.3)	1 (0.4)	2 (0.3)
Antiplatelet therapy			
Aspirin	446 (43.9)	130 (48.2)	316 (42.4)
P2Y12 inhibitors (e.g. clopidogrel, ticagrelor)	86 (8.5)	26 (9.6)	60 (8.0)
Digoxin	5 (0.5)	1 (0.4)	4 (0.5)
Diabetes management therapy			
Non-insulin antidiabetic agents^*^	910 (89.6)	238 (88.2)	672 (90.1)
Metformin	833 (82.0)	214 (79.3)	619 (83.0)
GLP-1RA	600 (59.1)	94 (34.8)	506 (67.8)
Liraglutide	333 (32.8)	47 (17.4)	286 (38.3)
Semaglutide	267 (26.3)	47 (17.4)	220 (29.5)
SGLT2-i	416 (40.9)	176 (65.2)	240 (32.2)
Canagliflozin	1 (0.1)	1 (0.4)	0 (0.0)
Empagliflozin	3 (0.3)	2 (0.7)	1 (0.1)
Dapagliflozin	412 (40.6)	173 (64.1)	239 (32.0)
Dipeptidyl peptidase-4 inhibitors	381 (37.5)	129 (47.8)	252 (33.8)
Sulfonylureas	315 (31.0)	110 (40.7)	205 (27.5)
Thiazolidinediones	38 (3.7)	12 (4.4)	26 (3.5)
Alpha-glucosidase inhibitors	10 (1.0)	3 (1.1)	7 (0.9)
Meglitinides	5 (0.5)	2 (0.7)	3 (0.4)
Insulin injections or pens^*^	554 (54.5)	126 (46.7)	428 (57.4)
Rapid-acting	358 (35.2)	84 (31.1)	274 (36.7)
Regular or short-acting	46 (4.5)	10 (3.7)	36 (4.8)
Intermediate-acting	45 (4.4)	6 (2.2)	39 (5.2)
Long-acting Insulin	409 (40.3)	102 (37.8)	307 (41.2)
Ultra-long-acting	41 (4.0)	3 (1.1)	38 (5.1)
Insulin Pump Use	4 (0.4)	2 (0.7)	2 (0.3)
None (New cases)	7 (0.7)	0 (0.0)	7 (0.9)

Numbers are presented as frequency (%) or mean ± SD.

* Some patients had multiple medications.

BMI, Body Mass Index; ACEIs, Angiotensin-Converting Enzyme Inhibitors; ARBs, Angiotensin II Receptor Blockers; CCBs, Calcium Channel Blocker; PCSK9, Proprotein Convertase Subtilisin/Kexin Type 9; P2Y12, purinergic receptor P2Y12; GLP-1RA, Glucagon-Like Peptide-1 Receptor Agonists; SGLT-2i, Sodium-Glucose Cotransporter-2 inhibitors.

### 3.3 Baseline clinical and laboratory parameters

At baseline, the overall mean body weight was 90.60 ± 18.9 kg, and the mean BMI was 34.71 ± 6.9 kg/m². Participants in the obese group had a significantly higher mean body weight (96.84 ± 17.4 kg) than those in the non-obese group (73.36 ± 10.1 kg). The mean baseline HbA1c for the overall cohort was 8.89 ± 1.8%, with comparable values across BMI groups. Baseline blood pressure and renal function were also similar between groups. Baseline values for all study outcomes are shown in **Table 3**.

**Table 3:** Baseline values for the study outcomes.

Characteristic	Overall	BMI group	
		BMI < 30	BMI ≥ 30
Weight, kg	90.60 ± 18.9	73.36 ± 10.1	96.84 ± 17.4
BMI, kg/m^2^	34.71 ± 6.9	27.02 ± 2.2	37.49 ± 5.8
Recent HbA1c, %	8.89 ± 1.8	8.96 ± 1.9	8.87 ± 1.8
Systolic BP, mmHg	134.73 ± 16.3	132.96 ± 16.1	135.38 ± 16.4
Diastolic BP, mmHg	71.74 ± 11.1	70.79 ± 10.2	72.09 ± 11.3
eGFR, ml/min/1.73m^2^	89.44 ± 25.4	86.59 ± 23.1	90.48 ± 26.1
Fasting BG, mmol/L	9.83 ± 3.8	10.07 ± 3.9	9.73 ± 3.8

Numbers are presented as mean ± SD.

BMI, Body Mass Index; HbA1c, Hemoglobin A1c (glycated hemoglobin); BP, Blood Pressure; eGFR, estimated Glomerular Filtration Rate; BG, Blood Glucose.

### 3.4 Change in weight and BMI over time

A progressive reduction in body weight was observed across all follow-up time points in the overall cohort. At 3 months, obese patients experienced significantly greater weight reduction compared to non-obese patients (−2.57 ± 4.8 kg vs −0.25 ± 3.8 kg, *p* < 0.0001). This difference remained evident at all follow-up time points (at 9 months: −3.00 ± 5.4 vs −0.86 ± 4.0 kg, *p* = 0.0002; and 12 months: −2.99 ± 8.0 kg vs −0.57 ± 3.7 kg, *p* = 0.0008). However, the difference between groups in the percentage change in body weight from baseline to each follow-up time point was only significant at the 3- and 6-month follow-ups. Reductions in BMI followed a similar pattern to weight change, with greater mean decreases observed in the obese group across all follow-up periods. These findings are detailed in **Table 4**.

**Table 4:** Mean change in weight, BMI and HbA1c from baseline to follow-ups.

Characteristic	Overall	BMI group		Between-group difference (95% confidence interval)	P-value
		BMI < 30	BMI ≥ 30		
Change in weight, kg					
At 3 months follow-up	-1.99 ± 4.7	-0.25 ± 3.8	-2.57 ± 4.8	-2.32 (-3.31, -1.33)	**<0.0001**
At 6 months follow-up	-1.97 ± 4.2	-1.07 ± 4.0	-2.30 ± 4.2	-1.23 (-2.12, -0.34)	**0.0069**
At 9 months follow-up	-2.45 ± 5.2	-0.86 ± 4.0	-3.00 ± 5.4	-2.15 (-3.24, -1.05)	**0.0002**
At 12 months follow-up	-2.30 ± 7.1	-0.57 ± 3.7	-2.99 ± 8.0	-2.42 (-3.84, -1.01)	**0.0008**
Percentage change in weight, %					
At 3 months follow-up	-1.85 ± 4.5	-0.63 ± 4.8	-2.29 ± 4.3	-1.65 (-2.35, -0.97)	**<0.0001**
At 6 months follow-up	-2.50 ± 5.8	-0.86 ± 5.3	-3.05 ± 5.9	-2.18 (-3.36, -1.01)	**0.0003**
At 9 months follow-up	-2.39 ± 7.7	-1.66 ± 5.9	-2.63 ± 8.1	-0.97 (-2.98, 1.04)	0.3402
At 12 months follow-up	-2.99 ± 5.9	-2.35 ± 7.1	-3.20 ± 5.5	-0.85 (-4.16, 2.46)	0.6109
Change in BMI, kg/m^2^					
At 3 months follow-up	-0.77 ± 1.8	-0.06 ± 1.4	-1.00 ± 1.9	-0.93 (-1.26, -0.61)	**<0.0001**
At 6 months follow-up	-0.76 ± 1.6	-0.39 ± 1.5	-0.89 ± 1.6	-0.50 (-0.84, -0.16)	**0.0040**
At 9 months follow-up	-0.96 ± 2.0	-0.31 ± 1.4	-1.19 ± 2.1	-0.88 (-1.28, -0.47)	**<0.0001**
At 12 months follow-up	-0.86 ± 2.9	-0.20 ± 1.3	-1.13 ± 3.3	-0.93 (-1.48, -0.37)	**0.0011**
Change in HbA1c, %					
At 3 months follow-up	-0.89 ± 1.6	-0.78 ± 1.7	-0.93 ± 1.5	-0.15 (-0.47, 0.17)	0.3614
At 6 months follow-up	-0.84 ± 1.5	-0.95 ± 1.7	-0.80 ± 1.5	0.15 (-0.16, 0.46)	0.3498
At 9 months follow-up	-1.07 ± 1.6	-0.92 ± 1.7	-1.12 ± 1.6	-0.20 (-0.58, 0.18)	0.3054
At 12 months follow-up	-0.82 ± 1.6	-0.56 ± 1.6	-0.92 ± 1.6	-0.36 (-0.76, 0.04)	0.0803
Change in fasting BG, mmol/L					
At 3 months follow-up	-1.39 ± 4.2	-1.13 ± 4.1	-1.49 ± 4.3	-0.36 (-1.59, 0.87)	0.5656
At 6 months follow-up	-1.37 ± 3.8	-1.43 ± 4.6	-1.34 ± 3.4	0.09 (-1.13, 1.31)	0.8692
At 9 months follow-up	-1.14 ± 4.6	-0.70 ± 5.7	-1.31 ± 4.1	-0.61 (-2.29, 1.07)	0.4719
At 12 months follow-up	-1.09 ± 3.5	-1.37 ± 3.2	-0.96 ± 3.6	0.41 (-1.62, 0.79)	0.5018

Numbers are presented as mean ± SD.

Between-group difference=(Obese Mean Change) − (Non-Obese Mean Change). A negative mean difference value indicates a greater reduction in the parameter (e.g., weight, BMI, or HbA1c) within the obese group compared to the non-obese group.

BMI, Body Mass Index; HbA1c, Hemoglobin A1c (glycated hemoglobin); BG, Blood Glucose.

Bold values indicate statistical significance.

As for the proportion of patients achieving at least a 5% reduction in weight as a clinically significant endpoint, obese patients demonstrated a statistically significant higher proportion of responders at the early and mid-term follow-up time points (3 and 6 months) compared to the non-obese cohort. By 9 and 12 months, the responder rates mathematically converged (partially due to the smaller sample size remaining at these later time points), but the early clinical success in the obese cohort remained evident. Both cohorts achieved meaningful glycemic reductions from their baselines. When restricted to patients who were uncontrolled at baseline (HbA1c ≥7.0%), the proportion of patients achieving the target of HbA1c <7.0% was comparable between cohorts at all intervals (e.g., Month 3: 16.2% vs. 13.9%, *p* = 0.4305). Similarly, the proportion of patients reaching robust HbA1c reduction thresholds (≥0.5% and ≥1.0%) did not differ significantly between the obese and non-obese groups (**Table 5**).

**Table 5:** Proportion of patients achieving clinical responder benchmarks^*^.

Characteristic	BMI group		*P*-value
	BMI < 30	BMI ≥ 30	
Weight (≥5% loss)			
At 3 months follow-up	26/216 (12.0%)	132/602 (21.9%)	**0.0016**
At 6 months follow-up	19/123 (15.5%)	100/368 (27.2%)	**0.0086**
At 9 months follow-up	14/55 (25.5%)	52/170 (30.6%)	0.4673
At 12 months follow-up	5/17 (29.4%)	16/51 (31.4%)	0.8795
HbA1c (≥0.5% reduction)			
At 3 months follow-up	127/242 (52.4%)	347/633 (54.8%)	0.5345
At 6 months follow-up	77/131 (58.8%)	252/384 (65.6%)	0.1589
At 9 months follow-up	38/62 (61.3%)	126/193 (65.3%)	0.5679
At 12 months follow-up	16/20 (80.0%)	35/56 (62.5%)	0.1528
HbA1c (≥1.0% reduction)			
At 3 months follow-up	85/242 (35.1%)	230/633 (36.3%)	0.7385
At 6 months follow-up	53/131 (40.5%)	183/384 (47.7%)	0.1533
At 9 months follow-up	27/62 (43.6%)	96/193 (49.7%)	0.3959
At 12 months follow-up	11/20 (55.0%)	27/56 (48.2%)	0.7950
HbA1c (achieving <7.0%)^**^			
At 3 months follow-up	30/216 (13.9%)	89/550 (16.2%)	0.4305
At 6 months follow-up	17/112 (15.2%)	78/341 (22.9%)	0.0826
At 9 months follow-up	12/55 (21.8%)	34/179 (19.0%)	0.6449
At 12 months follow-up	3/18 (16.7%)	6/51 (11.8%)	0.6874

Numbers are presented as frequency (%).

* Proportion of patients with reduction from baseline in weight by ≥5.0%, in HbA1c by ≥0.5% and ≥1.0%, and those reaching HbA1c <7.0% at each follow-up time point.

^**^ The analysis of HbA1c target achievement (<7.0%) was restricted exclusively to patients who presented with an uncontrolled baseline HbA1c (≥7.0%). Patients already at or below the clinical target at baseline were excluded from this specific outcome. BMI, Body Mass Index; HbA1c, Hemoglobin A1c (glycated hemoglobin).

Bold values indicate statistical significance.

### 3.5 Change in HbA1c and FBG over time

**Figure 2:**
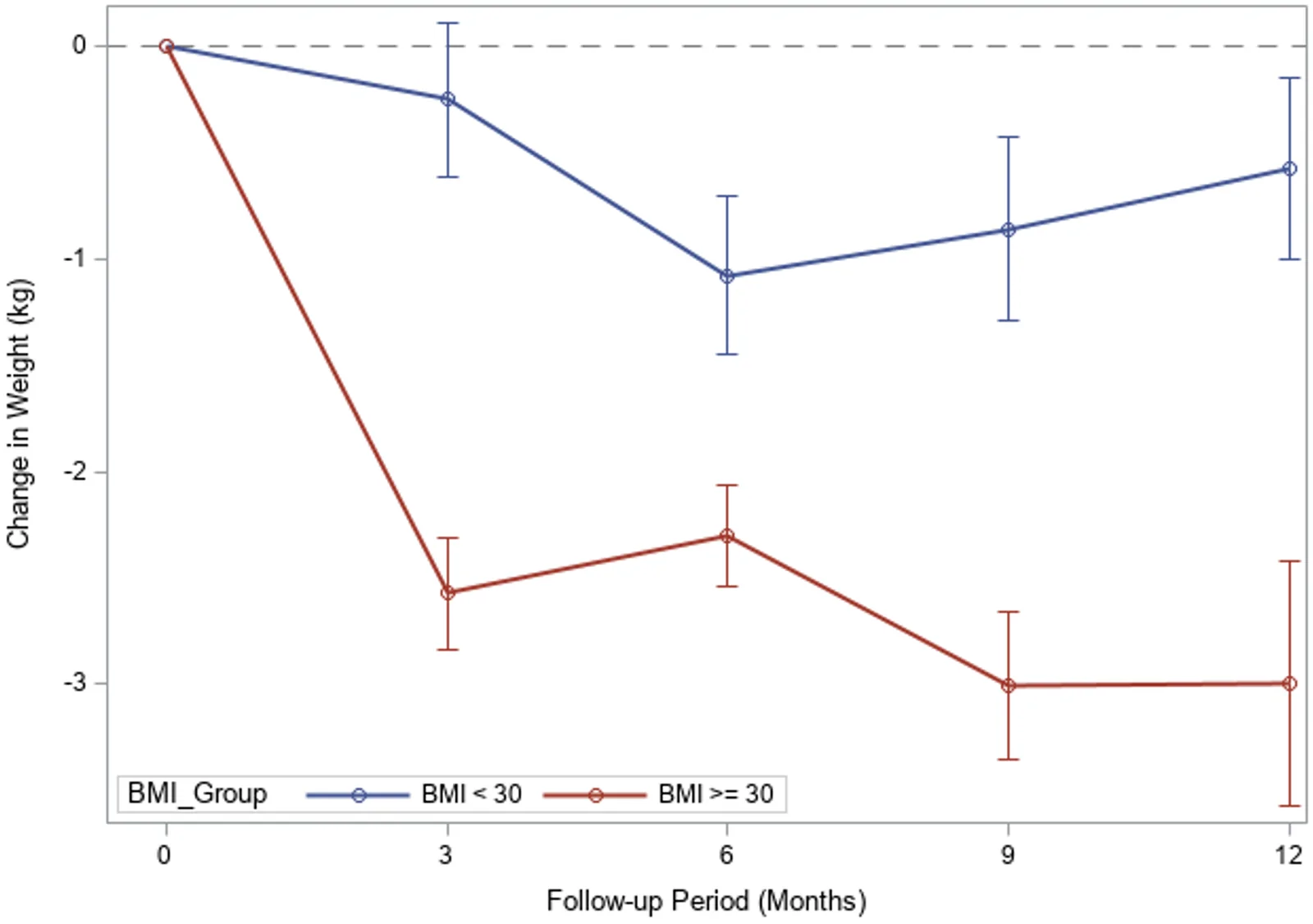
Mean change in weight (kg) from baseline to different follow-up periods, stratified by BMI.

**Figure 3:**
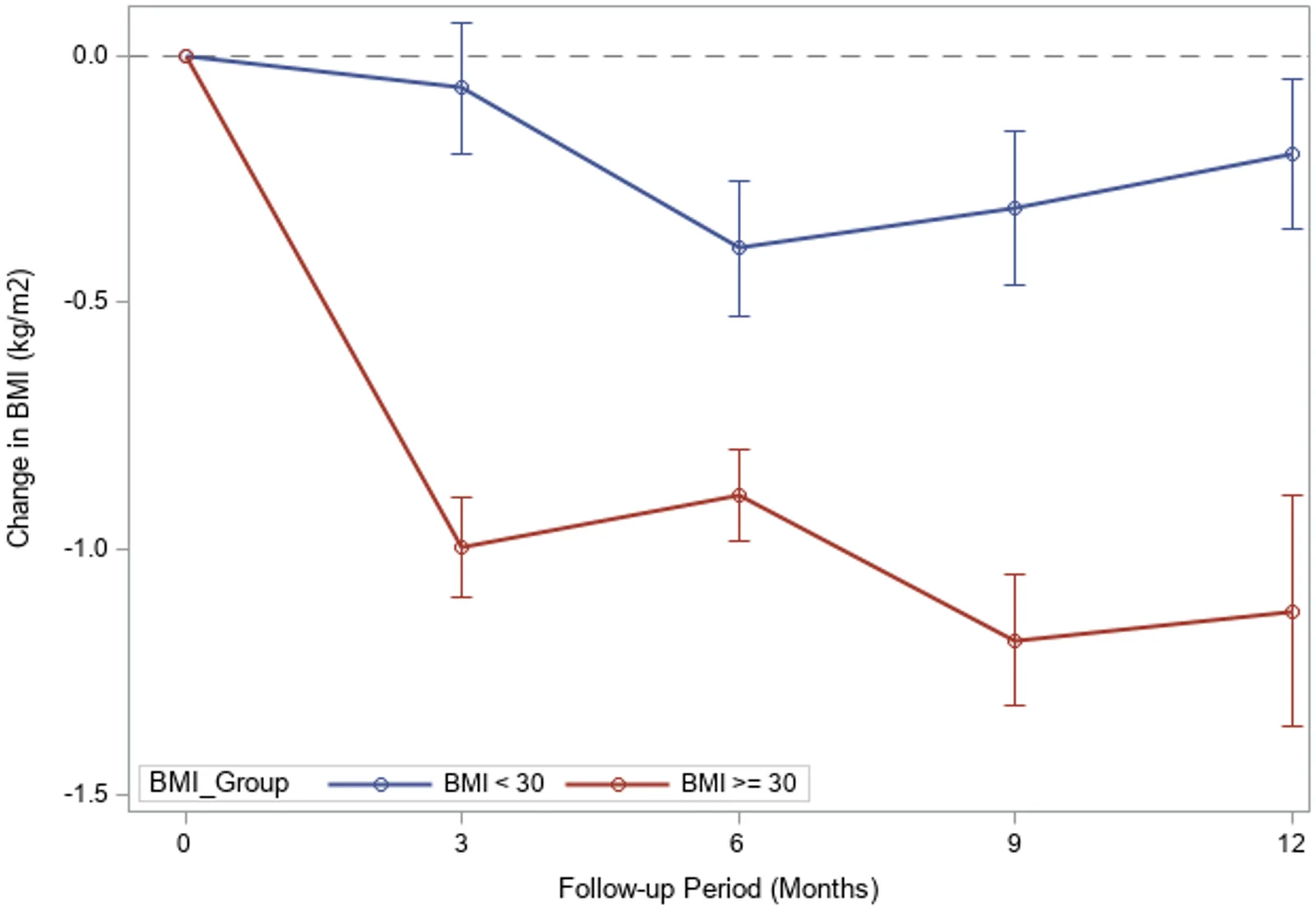
Mean change in BMI (kg/m^2^) from baseline to different follow-up periods, stratified by BMI.

**Figure 4:**
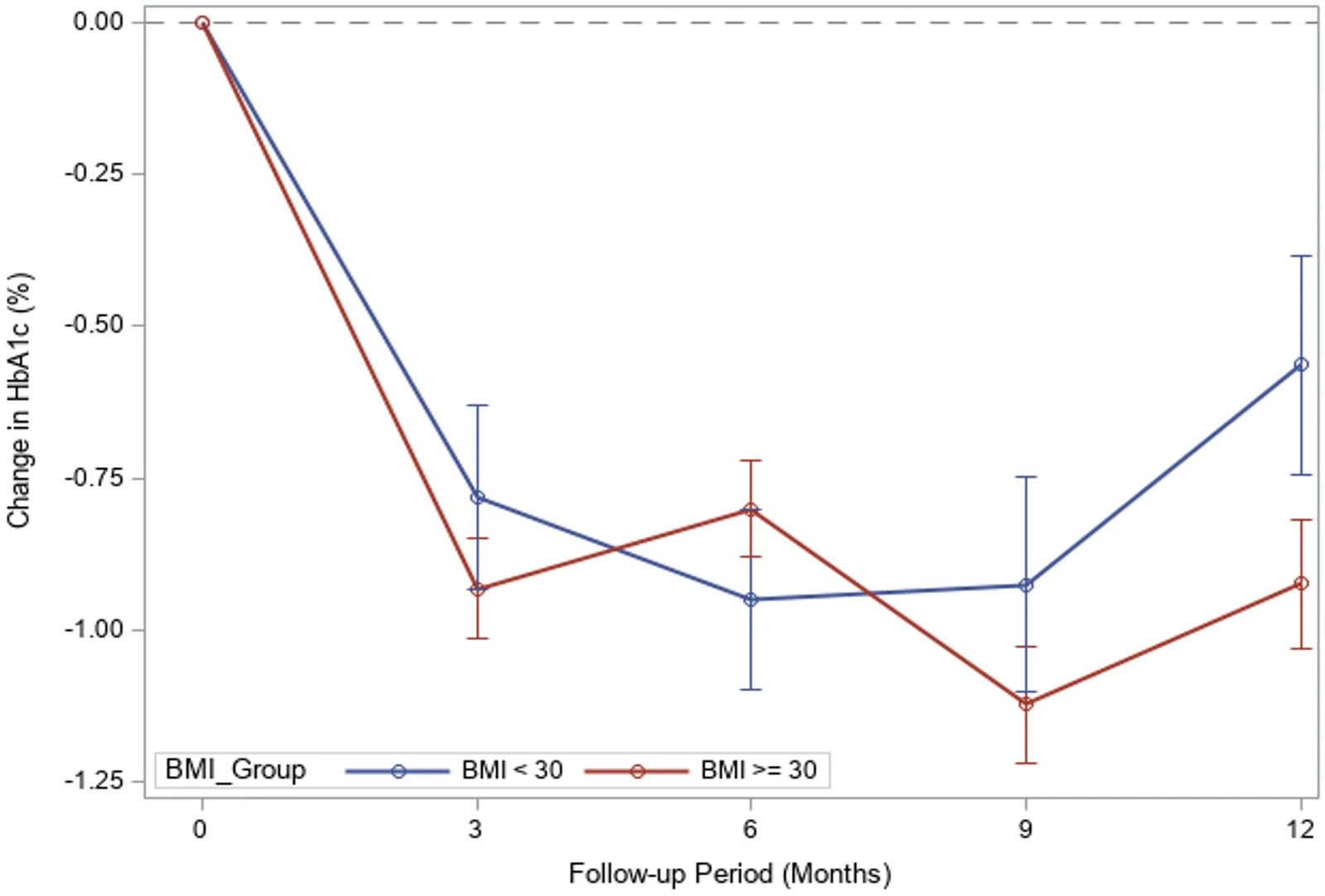
Mean change in HbA1c (%) from baseline to different follow-up periods, stratified by BMI.

Both BMI groups demonstrated clinically meaningful reductions in HbA1c across all follow-up periods. The mean HbA1c reduction for the overall cohort was −0.89 ± 1.6% at 3 months, −0.84 ± 1.5% at 6 months, −1.07 ± 1.6% at 9 months, and −0.82 ± 1.6% at 12 months. Likewise, reductions in FBG were observed across all follow-up periods in the overall cohort, with mean changes of −1.39 ± 4.2 mmol/L at 3 months, −1.37 ± 3.8 mmol/L at 6 months, −1.14 ± 4.6 mmol/L at 9 months, and −1.09 ± 3.5 mmol/L at 12 months. When comparing obese and non-obese patients, no statistically significant differences in HbA1c or FBG were observed at any follow-up time point (all *p*>0.05) (**Table 4**, **Figures 2**–**4**).

While the obese cohort consistently showed slightly higher percentages of patients achieving the HbA1c < 7.0% target during the first 6 months (e.g., 16.2% vs 13.9% at 3 months), the differences between the cohorts were not statistically significant at any time point (**Table 5**). These findings suggest that obesity status did not significantly influence the magnitude of HbA1c reduction.

### 3.6 Change in blood pressure, and renal function

Systolic blood pressure showed small reductions over time (−2.40 ± 16.1, −2.69 ± 15.8, −3.79 ± 17.9, and −2.50 ± 15.8 mmHg at 3, 6, 9, and 12 months, respectively), while diastolic blood pressure demonstrated minimal changes. No significant between-group differences were detected at any follow-up period. Similarly, renal function, assessed by eGFR, remained largely unchanged throughout the follow-up periods, with mean changes close to zero at all time points and no significant differences between BMI groups (**Table 6**).

**Table 6:** Mean change in blood pressure and eGFR from baseline to follow-ups.

Characteristic	Overall	BMI group		Between-group difference (95% confidence interval)	*P*-value
		BMI < 30	BMI ≥ 30		
Change in systolic BP, mmHg					
At 3 months follow-up	-2.40 ± 16.1	-2.56 ± 15.1	-2.35 ± 16.4	0.21 (-3.14, 3.57)	0.9004
At 6 months follow-up	-2.69 ± 15.8	-3.49 ± 16.8	-2.39 ± 15.4	1.10 (-2.15, 4.35)	0.5057
At 9 months follow-up	-3.79 ± 17.9	-4.77 ± 18.1	-3.44 ± 17.8	1.33 (-2.93, 5.59)	0.5394
At 12 months follow-up	-2.50 ± 15.8	-1.73 ± 15.6	-2.79 ± 15.9	-1.06 (-5.11, 2.99)	0.6059
Change in diastolic BP, mmHg					
At 3 months follow-up	0.08 ± 10.5	-1.19 ± 10.2	0.48 ± 10.6	1.67 (-0.52, 3.86)	0.1348
At 6 months follow-up	-0.26 ± 10.6	-1.35 ± 10.2	0.15 ± 10.7	1.49 (-0.68, 3.66)	0.1771
At 9 months follow-up	-0.95 ± 11.4	-1.89 ± 10.4	-0.61 ± 11.8	1.28 (-1.44, 4.00)	0.3541
At 12 months follow-up	-0.77 ± 10.2	-0.99 ± 10.0	-0.68 ± 10.3	0.31 (-2.31, 2.92)	0.8184
Change in eGFR, ml/min/1.73m^2^					
At 3 months follow-up	-0.68 ± 15.7	-0.20 ± 14.4	-0.86 ± 16.3	-0.66 (-4.17, 2.86)	0.7140
At 6 months follow-up	-0.56 ± 14.4	1.06 ± 11.8	-1.21 ± 15.3	-2.28 (-5.11, 0.56)	0.1155
At 9 months follow-up	-0.78 ± 15.0	1.46 ± 13.7	-1.57 ± 15.3	-3.04 (-6.76, 0.69)	0.1098
At 12 months follow-up	-2.41 ± 15.7	-1.32 ± 12.9	-2.85 ± 16.7	-1.53 (-5.34, 2.28)	0.4783

Numbers are presented as mean ± SD.

Between-group difference=(Obese Mean Change) − (Non-Obese Mean Change). A negative mean difference value indicates a greater reduction in the parameter (e.g., systolic BP, diastolic BP, or eGFR) within the obese group compared to the non-obese group.

BMI, Body Mass Index; BP, Blood Pressure; eGFR, estimated Glomerular Filtration Rate.

### 3.7 Sub-group analysis and multivariate adjustment

To further evaluate whether therapeutic choice influenced metabolic response, we conducted a subgroup analysis stratified by medication class (GLP-1 RA vs. SGLT-2i). In the obese cohort, GLP-1 RA therapy was associated with significantly greater weight reduction at 3, 9, and 12 months compared to SGLT-2i therapy (p < 0.05). Similarly, BMI reductions remained more pronounced in the GLP-1 RA sub-group within the obese cohort. In the non-obese cohort, metabolic improvements were comparable between the two medication classes across all time points. These supplemental findings (**Supplementary Tables 1**, **S2**) suggest that while both medication classes are effective, the weight-management benefit of GLP-1 RA may be more pronounced in patients with a higher baseline BMI. Additionally, when evaluating each medication class individually within our cohort, significant differences in weight reduction were observed between obese and non-obese patients receiving GLP-1RA at 3 and 12 months, and among those receiving SGLT-2i at 6 and 9 months, with greater weight reduction observed in the obese cohort (**Supplementary Table 3**).

To account for the influence of baseline clinical confounders and to evaluate the robustness of our findings, we performed a mixed-effects model for repeated measures (MMRM) adjusted for clinical confounders and concurrent medications. When comparing unadjusted longitudinal changes with those derived from the fully adjusted MMRM (**Table 7**), the unadjusted raw mean changes (**Tables 4**, **6**) demonstrated pronounced differences in weight and BMI, the fully adjusted MMRM provided more precise estimates of the metabolic impact. The obese cohort consistently demonstrated significantly greater absolute weight and BMI reductions compared to the non-obese cohort; these differences remained robust and statistically significant after adjusting for baseline demographic and clinical covariates. Crucially, when evaluating relative percentage weight change (%) in the adjusted MMRM framework—which accounts for baseline body mass disparities—the obese cohort achieved significantly greater adjusted relative weight loss across all follow-up intervals. While glycemic metrics (HbA1c and FBG) improved in both cohorts from baseline, the apparent between-group differences observed in raw data were further attenuated and verified in the adjusted model, indicating that baseline population differences influenced the observed variation in glycemic response as well.

**Table 7:** Multivariable-adjusted MMRM estimates of metabolic changes from baseline stratified by BMI.

Characteristic	BMI group		Between-group difference (95% confidence interval)	*P*-value
	BMI < 30	BMI ≥ 30		
Change in weight, kg				
At 3 months follow-up	-0.41 ± 0.51 (n=216)	-2.44 ± 0.31 (n=602)	**-2.03 (-3.24, -0.81)**	**0.0011**
At 6 months follow-up	-0.87 ± 0.47 (n=123)	-2.16 ± 0.30 (n=368)	**-1.29 (-2.42, -0.16)**	**0.0251**
At 9 months follow-up	-0.86 ± 0.54 (n=55)	-3.08 ± 0.33 (n=170)	**-2.22 (-3.48, -0.96)**	**0.0007**
At 12 months follow-up	-1.04 ± 0.58 (n=17)	-2.89 ± 0.38 (n=51)	**-1.85 (-3.24, -0.46)**	**0.0094**
Percentage change in weight, %				
At 3 months follow-up	-0.25 ± 0.61 (n=216)	-2.64 ± 0.35 (n=602)	**-2.38 (-3.89, -0.87)**	**0.0020**
At 6 months follow-up	-0.84 ± 0.57 (n=123)	-2.43 ± 0.35 (n=368)	**-1.58 (-3.00, -0.16)**	**0.0287**
At 9 months follow-up	-0.86 ± 0.64 (n=55)	-3.30 ± 0.37 (n=170)	**-2.43 (-4.00, -0.87)**	**0.0023**
At 12 months follow-up	-1.02 ± 0.68 (n=17)	-3.15 ± 0.42 (n=51)	**-2.13 (-3.80, -0.46)**	**0.0126**
Change in BMI, kg/m^2^				
At 3 months follow-up	-0.25 ± 0.14 (n=216)	-0.79 ± 0.08 (n=602)	**-0.55 (-0.88, -0.21)**	**0.0014**
At 6 months follow-up	-0.35 ± 0.14 (n=123)	-1.03 ± 0.08 (n=368)	**-0.68 (-1.02, -0.34)**	**<0.0001**
At 9 months follow-up	-0.38 ± 0.15 (n=55)	-1.04 ± 0.09 (n=170)	**-0.66 (-1.02, -0.30)**	**0.0003**
At 12 months follow-up	-0.74 ± 0.17 (n=17)	-0.98 ± 0.10 (n=51)	-0.24 (-0.63, 0.16)	0.2360
Change in HbA1c, %				
At 3 months follow-up	-0.66 ± 0.14 (n=242)	-0.72 ± 0.09 (n=633)	-0.07 (-0.40, 0.27)	0.7009
At 6 months follow-up	-0.96 ± 0.13 (n=131)	-1.04 ± 0.08 (n=384)	-0.08 (-0.40, 0.23)	0.6071
At 9 months follow-up	-0.75 ± 0.15 (n=62)	-1.03 ± 0.09 (n=193)	-0.28 (-0.63, 0.08)	0.1281
At 12 months follow-up	-0.76 ± 0.16 (n=20)	-1.01 ± 0.10 (n=56)	-0.24 (-0.63, 0.14)	0.2159
Change in fasting BG, mmol/L				
At 3 months follow-up	-0.69 ± 0.44 (n=242)	-1.37 ± 0.27 (n=633)	-0.68 (-1.72, 0.36)	0.1993
At 6 months follow-up	-1.48 ± 0.41 (n=131)	-1.20 ± 0.27 (n=384)	0.28 (-1.28, 0.71)	0.5791
At 9 months follow-up	0.22 ± 0.48 (n=62)	-1.30 ± 0.29 (n=193)	**-1.52 (-2.65, -0.39)**	**0.0084**
At 12 months follow-up	-1.11 ± 0.48 (n=20)	-1.56 ± 0.35 (n=56)	-0.45 (-1.64, 0.74)	0.4566

MMRM, Mixed-Effects Model for Repeated Measures.

Data are presented as adjusted least square mean (LSM) change from baseline (± SE). Differences are presented as LSM difference [95% CI]. Models were adjusted for age, gender, HbA1c, eGFR, SBP, DBP, smoking, comorbidities, and concurrent medication use.

Between-group comparison reflects the LSM difference derived from the adjusted MMRM:.

LSM_difference_=LSM_Obese_ - LSM_Non-Obese_; A negative LSM difference indicates a greater adjusted mean reduction in the parameter (e.g., weight, BMI, or HbA1c) in the obese group relative to the non-obese group after controlling for baseline covariates.

BMI, Body Mass Index; HbA1c, Hemoglobin A1c (glycated hemoglobin); BG, Blood Glucose.

Bold values indicate statistical significance.

## 4 Discussion

This multicenter retrospective study evaluated whether baseline obesity status was associated with differences in glycemic and weight responses to contemporary glucose-lowering therapies, specifically SGLT-2i and GLP-1RA, in adults with T2DM. By stratifying patients according to BMI and examining longitudinal changes over 12 months, this study provides real-world evidence regarding the association between baseline obesity status and therapeutic outcomes in routine clinical practice. We observed that obese patients were associated with greater reductions in absolute body weight after multivariable adjustment, although their higher baseline body weight may have contributed to these differences. We did not observe that obesity status was associated with differential HbA1c reduction. However, residual confounding, treatment selection bias, and follow-up attrition limit causal interpretation. The greater weight reduction observed among obese individuals is biologically plausible and consistent with the mechanisms of action of both drug classes. GLP-1RA reduce caloric intake through appetite suppression and delayed gastric emptying, while SGLT-2i induce caloric loss through glucosuria. However, given the retrospective observational design, these findings should not be interpreted as demonstrating that obesity directly modifies treatment response. Rather, they generate the hypothesis that baseline BMI may be associated with differential weight outcomes following treatment with GLP-1RA and SGLT-2i. The more modest weight and BMI reductions observed in our study likely reflect the heterogeneity of real-world clinical practice, differences in patient characteristics (including higher baseline body weight in the obese group), and the greater use of liraglutide compared with semaglutide, particularly among obese patients.

In contrast, HbA1c reduction was similar between BMI groups despite the difference in weight loss. This is a clinically important observation because obesity is closely linked to insulin resistance, and greater weight loss would traditionally be expected to produce greater glycemic benefit. The comparable HbA1c reductions suggest that the glucose-lowering effects of GLP-1RA and SGLT-2i are largely independent of baseline adiposity and primarily driven by their pharmacologic mechanisms. Similar observations have been reported in network meta-analyses where HbA1c response was not strongly predicted by baseline BMI in patients treated with these agents (9). This supports the concept that these therapies exert glycemic effects that extend beyond weight-mediated pathways. Additionally, dose intensity and escalation data were not available in our study, limiting our ability to determine whether patients were receiving the recommended maintenance doses. However, this limitation reflects real-world practice, where challenges may exist in ensuring that patients receive and maintain the recommended doses (16).

The modest reductions in blood pressure and the stability of renal function observed across both BMI groups are also consistent with the cardiorenal protective profiles demonstrated in major outcome trials of GLP-1RA and SGLT-2i. Cardiovascular and renal outcome studies have consistently shown that the benefits of SGLT-2i extend beyond glycemic control (17–19). Similarly, GLP-1RA have demonstrated cardiovascular protection regardless of baseline BMI in clinical trials (20).

In a real-world prospective study evaluating liraglutide, obese and non-obese patients with T2DM were compared over 18 months (11). The study demonstrated that while both groups experienced significant reductions in HbA1c, obese patients showed a greater decrease compared to non-obese patients (-1.8% vs. -1.2%, *p* < 0.05). Additionally, weight loss was more pronounced in obese individuals (-7.2 kg vs. -3.9 kg, *p* < 0.01). Improvements in lipid profiles, blood pressure, and inflammatory markers were also greater in obese patients. These findings suggest that baseline BMI influences the magnitude of response to liraglutide, with obese patients deriving greater glycemic and cardiometabolic benefits compared to non-obese individuals (11).

Another prospective study assessed the effects of empagliflozin, an SGLT-2i, in obese and non-obese patients with T2DM over 24 weeks (12). Both groups demonstrated significant reductions in HbA1c levels, with the obese group decreasing from 8.5% to 7.2% and the non-obese group from 8.3% to 7.1%. In terms of weight reduction, obese patients experienced a greater average weight loss compared to non-obese patients (-5.4 kg vs. -2.8 kg). Although glycemic improvements were observed in both groups, the magnitude of weight loss was more substantial in obese individuals. These findings indicate that empagliflozin is effective across BMI categories, with enhanced weight reduction in obese patients, while HbA1c reduction remained comparable between groups (12). The present study adds real-world evidence suggesting an association between baseline BMI and the magnitude of weight reduction achieved with these therapies in routine clinical practice.

It is worth mentioning that the efficacy of SGLT-2i in lowering HbA1c is dependent on kidney function, and diminishes significantly as kidney function declines (21). However, their cardiovascular and renal protective benefits remain robust even in advanced chronic kidney disease (CKD), making them highly recommended regardless of their glucose- lowering effect (21). As these drugs act by inhibiting glucose reabsorption in the proximal renal tubules, their glucose-lowering effect is directly dependent on the filtered glucose load, which is determined by eGFR (22). The impact of this factor on our findings is likely minimal as the prevalence of CKD at baseline was low (7.5% across the entire cohort), with advanced CKD (stages 4–5) comprising only 1.2% of the study population. Additionally, changes in eGFR across the follow-up periods were not statistically significant indicating that SGLT-2i activity was theoretically maintained across the study period. Furthermore, the limited availability of albuminuria data due to the retrospective nature of the study may have limited the appropriate assessment of kidney disease and treatment response. However, a recent meta-analysis demonstrated consistent benefits of SGLT-2i on kidney, hospitalization, and mortality outcomes irrespective of diabetes status or urinary albumin-to-creatinine ratio (UACR) (23).

Moreover, important pharmacological differences exist between semaglutide and liraglutide, rendering them not interchangeable. Liraglutide was the first long-acting GLP-1 analogue, achieved by attaching a C16 fatty acid that enables albumin binding and extends its half-life from about 2 minutes to 13–15 hours, making once-daily dosing possible (24). Semaglutide further improved this design by using a longer C18 fatty diacid for stronger albumin binding and adding an aminoisobutyric acid (Aib) substitution at position 2, which makes it highly resistant to DPP-4 degradation. These changes extend its half-life to about seven days, allowing once-weekly dosing, while also increasing its potency at GLP-1 receptors, leading to stronger effects on appetite, insulin secretion, and weight loss than liraglutide. These pharmacological advantages have resulted in superior efficacy for reducing both HbA1c and body weight in clinical trials (9). In our cohort, liraglutide was prescribed more frequently than semaglutide, particularly among obese patients (38.3% vs. 29.5%), which may have contributed to the relatively smaller reductions in body weight observed in this group. Additionally, the utilization of DPP-4 inhibitors was higher in the non-obese group (47.8% vs. 33.8%) which may have enhanced the observed treatment effect of GLP-1 RA. In contrast, empagliflozin has the highest selectivity for SGLT-2 relative to SGLT-1 (approximately 2,500-fold), followed by dapagliflozin (approximately 1,200-fold), while canagliflozin is the least selective (approximately 250-fold) (25, 26). These pharmacodynamic differences indicate that SGLT-2i should not be viewed as pharmacologically equivalent. Therefore, heterogeneity within the GLP-1RA and SGLT-2i classes may have contributed to variability in the observed outcomes in this study.

Baseline insulin utilization in our cohort was higher in the obese group than in the non-obese group (57.4% vs. 46.7%), which may have contributed to the more modest reduction in body weight observed in the obese group. Insulin utilization may offset weight loss by promoting lipogenesis and preventing the breakdown of existing adipose tissue (27, 28). Baseline insulin use may also represent a surrogate marker of longer diabetes duration, more advanced β-cell dysfunction, or greater disease severity. These factors could independently influence both glycemic response and weight trajectories and therefore should be recognized as potential residual confounders. Additionally, the greater baseline body weight of our cohort may explain the larger absolute weight loss observed, as individuals with higher body weight generally have higher resting metabolic rates and total energy expenditure, resulting in larger absolute energy deficits (28). Moreover, greater adiposity is associated with increased insulin resistance, making excess body fat an important determinant of both weight and metabolic health. The greater weight reduction observed among obese patients may also be explained by obesity-related metabolic adaptations. Weight loss has been associated with reductions in adipose tissue inflammation and ectopic fat accumulation, as well as improvements in hepatic insulin sensitivity, all of which contribute to improved metabolic health. However, these physiological changes do not necessarily result in greater HbA1c reduction, as glycemic response is influenced by multiple factors beyond weight loss alone, including baseline glycemic status, residual pancreatic β-cell function, duration of diabetes, and the type of therapeutic intervention (29). Consequently, improvements in HbA1c do not always parallel changes in body weight. Individuals with similar degrees of weight loss may experience markedly different reductions in HbA1c because glycemic improvement reflects not only the loss of adiposity but also the extent to which insulin resistance and glucose metabolism improve.

Notably, the baseline prevalence of key comorbidities, including CKD, heart failure, hypertension, ASCVD, and obesity, was comparable to that reported previously among patients with T2DM in SA (30). This similarity suggests that our study cohort is representative of the broader Saudi T2DM population encountered in routine clinical practice, thereby enhancing the external validity and generalizability of our findings. However, extrapolation of these findings to other ethnic groups and healthcare systems should remain cautious, because prescribing practices, obesity prevalence, healthcare access, and metabolic characteristics vary substantially across different populations.

From a clinical perspective, current diabetes guidelines recommend the use of GLP-1RA or SGLT-2i particularly in patients who are overweight or obese, largely due to their favorable effects on body weight (31). The findings of this study suggest an association between obesity and greater weight reduction, while glycemic improvements were broadly similar across BMI categories. Accordingly, patients with obesity may experience more noticeable weight-related benefits when this is a primary therapeutic goal. At the same time, our findings did not provide strong evidence that obesity status influenced the magnitude of HbA1c reduction suggesting that obesity status may not be the sole factor to consider when selecting therapy for glycemic control. Our findings should be interpreted as associations observed in routine clinical practice rather than evidence of causal treatment effects and that it should be considered as hypothesis-generating observation. Thus, future studies should further explore the interaction between BMI, weight change, and glycemic response to optimize individualized diabetes management. Despite multivariable adjustment, residual confounding inherent to the retrospective observational design cannot be eliminated. Additionally, the baseline treatment imbalance between obese and non-obese patients may have influenced the observed treatment response; therefore, the findings should be interpreted as associations rather than evidence of a causal modifying effect of obesity.

This study has several limitations. Its retrospective observational design and non-randomized treatment allocation limit causal inference and introduce potential selection bias and confounding by indication. Importantly, treatment selection may have been influenced by baseline patient characteristics, including obesity status, glycemic control, comorbidities, concomitant medications, and clinician treatment preferences, which may also have influenced subsequent weight and glycemic outcomes. Although multivariable adjustment was performed, residual confounding may persist, particularly because lifestyle interventions, adherence, dose intensity and escalation, treatment discontinuation, changes in background therapy, and other interventions were not fully captured. Thus, the observed differences in weight and glycemic outcomes should be interpreted as associations rather than evidence that baseline obesity status causally modifies treatment response. Incomplete documentation of baseline variables and heterogeneity among individual agents within each therapeutic class may also have contributed to variability in treatment response. Additionally, substantial follow-up attrition occurred; although MMRM accounts for missing data under the missing-at-random assumption, we did not perform a formal sensitivity analysis to assess potential informative censoring. While our unadjusted analysis demonstrated that unadjusted percentage weight loss attenuated at 9 and 12 months in raw data (**Table 4**), our fully adjusted longitudinal MMRM model specifically evaluated percentage weight change (%) and confirmed that baseline obesity was associated with significantly greater relative weight loss across all 12 months after controlling for baseline mass, comorbidities, and concomitant medications (**Table 7**). Nevertheless, residual confounding inherent to retrospective database studies—including uncaptured variations in lifestyle modifications, dietary adherence, and exact medication titration schedules—cannot be completely ruled out.

Despite these limitations, important strengths include a large sample size across three major centers and the use of standardized follow-up windows, which allowed consistent longitudinal comparison. Another key strength of this study is its preservation of real-world clinical validity. Rather than utilizing matching algorithms that routinely discard representative patients to balance highly skewed treatment allocations, our multivariable modeling approach allowed us to fully adjust for the differences in baseline pharmacotherapy and baseline characteristics while maintaining the complete intention-to-treat observational cohort.

## 5 Conclusion

In adults with T2DM treated with SGLT-2i or GLP-1RA, we observed an association between obesity status and reductions in absolute body weight and BMI, but not between obesity status and HbA1c reduction after multivariable adjustment. However, the obese group had higher baseline body weight, which may have contributed to these differences. Although these real-world observations provide clinically relevant insights, they should be considered hypothesis-generating rather than definitive because of the retrospective observational design. Further prospective studies are needed to confirm whether baseline BMI modifies treatment response and to inform treatment decisions based on therapeutic goals, including weight management.

## Data Availability

The datasets used and/or analyzed during the current study are available from the corresponding author on reasonable request. Requests to access these datasets should be directed to omaraalshaya@gmail.com.

## References

[1] Klein S Gastaldelli A Yki-Järvinen H Scherer PE . Why does obesity cause diabetes? Cell Metab. (2022) 34:11–20. doi: 10.1016/j.cmet.2021.12.012 34986330 PMC8740746

[2] Ferrannini E . Sodium-glucose co-transporters and their inhibition: Clinical physiology. Cell Metab. (2017) 26:27–38. doi: 10.1016/j.cmet.2017.04.011 28506519

[3] Wright EM . SGLT2 inhibitors: Physiology and pharmacology. Kidney360. (2021) 2:2027–37. doi: 10.34067/kid.0002772021 35419546 PMC8986039

[4] Pereira MJ Eriksson JW . Emerging role of SGLT-2 inhibitors for the treatment of obesity. Drugs. (2019) 79:219–30. doi: 10.1007/s40265-019-1057-0 30701480 PMC6394798

[5] Pharmacologic approaches to glycemic treatment: Standards of care in diabetes-2026. Diabetes Care. (2026) 49:S183–215. doi: 10.2337/dc26-s009 41358900 PMC12690185

[6] Davies MJ Aroda VR Collins BS Gabbay RA Green J Maruthur NM . Management of hyperglycaemia in type 2 diabetes, 2022. A consensus report by the American Diabetes Association (ADA) and the European Association for the Study of Diabetes (EASD). Diabetologia. (2022) 65:1925–66. doi: 10.2337/figshare.20800537 PMC9510507 36151309

[7] Yeh TL Tsai MC Tsai WH Tu YK Chien KL . Effect of glucagon-like peptide-1 receptor agonists on glycemic control, and weight reduction in adults: A multivariate meta-analysis. PLoS One. (2023) 18:e0278685. doi: 10.1371/journal.pone.0278685 36696429 PMC9876280

[8] Raza FA Altaf R Bashir T Asghar F Altaf R Tousif S . Effect of GLP-1 receptor agonists on weight and cardiovascular outcomes: A review. Med (Baltimore). (2024) 103:e40364. doi: 10.1097/md.0000000000040364 39496023 PMC11537668

[9] Ma H Lin Y-H Dai L-Z Lin C-S Huang Y Liu S-Y . Efficacy and safety of GLP-1 receptor agonists versus SGLT-2 inhibitors in overweight/obese patients with or without diabetes mellitus: a systematic review and network meta-analysis. BMJ Open. (2023) 13:e061807. doi: 10.1136/bmjopen-2022-061807 36882248 PMC10008474

[10] Cai X Ji L Chen Y Yang W Zhou L Han X . Comparisons of weight changes between sodium-glucose cotransporter 2 inhibitors treatment and glucagon-like peptide-1 analogs treatment in type 2 diabetes patients: A meta-analysis. J Diabetes Investig. (2017) 8:510–7. doi: 10.1111/jdi.12625 28106956 PMC5497054

[11] Nikolic D Patti AM Giglio RV Chianetta R Castellino G Magán-Fernández A . Liraglutide improved cardiometabolic parameters more in obese than in non-obese patients with type 2 diabetes: A real-world 18-month prospective study. Diabetes Ther. (2022) 13:453–64. doi: 10.1007/s13300-022-01217-z 35167051 PMC8853434

[12] Dedemen B Duman TT Dedemen MM Aktas G . Effect of sodium glucose co-transporter 2 inhibitor use on anthropometric measurements and blood glucose in obese and non-obese type 2 diabetic patients. Clin Nutr ESPEN. (2024) 63:515–9. doi: 10.1016/j.clnesp.2024.07.016 39047870

[13] Montanya E Fonseca V Colagiuri S Blonde L Donsmark M Nauck MA . Improvement in glycated haemoglobin evaluated by baseline body mass index: a meta-analysis of the liraglutide phase III clinical trial programme. Diabetes Obes Metab. (2016) 18:707–10. doi: 10.1111/dom.12617 26662611 PMC5067695

[14] Wilding JPH Batterham RL Calanna S Davies M Van Gaal LF Lingvay I . Once-weekly semaglutide in adults with overweight or obesity. N Engl J Med. (2021) 384:989–1002. doi: 10.1056/nejmoa2032183 33567185

[15] Zinman B Wanner C Lachin JM Fitchett D Bluhmki E Hantel S . Empagliflozin, cardiovascular outcomes, and mortality in type 2 diabetes. N Engl J Med. (2015) 373:2117–28. doi: 10.1056/nejmoa1504720 26378978

[16] Armstrong S Mostovoy L Gaich G Noor MA . 758-P: Titration to maintenance doses of GLP-1R agonists for overweight and obesity is suboptimal for majority of patients—evidence from a real-world claims dataset. Diabetes. (2025) 74. doi: 10.2337/db25-758-p

[17] Marso SP Daniels GH Brown-Frandsen K Kristensen P Mann JF Nauck MA . Liraglutide and cardiovascular outcomes in type 2 diabetes. N Engl J Med. (2016) 375:311–22. doi: 10.1056/nejmoa1603827 27295427 PMC4985288

[18] Neal B Perkovic V Matthews DR . Canagliflozin and cardiovascular and renal events in type 2 diabetes. N Engl J Med. (2017) 377:2099. doi: 10.1056/nejmoa1611925 29166232

[19] Wiviott SD Raz I Bonaca MP Mosenzon O Kato ET Cahn A . Dapagliflozin and cardiovascular outcomes in type 2 diabetes. N Engl J Med. (2019) 380:347–57. doi: 10.1056/nejmoa1812389 30415602

[20] Marso SP Bain SC Consoli A Eliaschewitz FG Jódar E Leiter LA . Semaglutide and cardiovascular outcomes in patients with type 2 diabetes. N Engl J Med. (2016) 375:1834–44. doi: 10.1056/nejmoa1607141 27633186

[21] Kelly MS Lewis J Huntsberry AM Dea L Portillo I . Efficacy and renal outcomes of SGLT2 inhibitors in patients with type 2 diabetes and chronic kidney disease. Postgrad Med. (2019) 131:31–42. doi: 10.1080/00325481.2019.1549459 30449220

[22] Nespoux J Vallon V . Renal effects of SGLT2 inhibitors: an update. Curr Opin Nephrol Hypertens. (2020) 29:190–8. doi: 10.1097/mnh.0000000000000584 PMC7224333 31815757

[23] Staplin N Roddick AJ Neuen BL Anker SD Bhatt DL Butler J . Effects of sodium glucose cotransporter 2 inhibitors by diabetes status and level of albuminuria: A meta-analysis. Jama. (2026) 335:220–32. doi: 10.1001/jama.2025.20835 41202026 PMC12595550

[24] Knudsen LB Lau J . The discovery and development of liraglutide and semaglutide. Front Endocrinol (Lausanne). (2019) 10:155. doi: 10.3389/fendo.2019.00155 31031702 PMC6474072

[25] Williams DM Nawaz A Evans M . Sodium-glucose co-transporter 2 (SGLT2) inhibitors: Are they all the same? A narrative review of cardiovascular outcome trials. Diabetes Ther. (2021) 12:55–70. doi: 10.1007/s13300-020-00951-6 33185854 PMC7843788

[26] Hsia DS Grove O Cefalu WT . An update on sodium-glucose co-transporter-2 inhibitors for the treatment of diabetes mellitus. Curr Opin Endocrinol Diabetes Obes. (2017) 24:73–9. doi: 10.1097/med.0000000000000311 27898586 PMC6028052

[27] Apovian CM Okemah J O'Neil PM . Body weight considerations in the management of type 2 diabetes. Adv Ther. (2019) 36:44–58. doi: 10.1007/s12325-018-0824-8 30465123 PMC6318231

[28] Theodorakis N Kreouzi M Pappas A Nikolaou M . Beyond calories: Individual metabolic and hormonal adaptations driving variability in weight management-a state-of-the-art narrative review. Int J Mol Sci. (2024) 25:13438. doi: 10.3390/ijms252413438 39769203 PMC11676201

[29] Palmer R Neiberg RH Beavers KM Kahn SE Houston DK Wagenknecht L . Associations between decreases in adiposity and reductions in HbA1c and insulin use in the Look AHEAD cohort. Obes (Silver Spring). (2025) 33:612–20. doi: 10.1002/oby.24242 39904719 PMC11897852

[30] Alshaya OA Korayem GB Alghwainm M Alyami W Alotaibi A Alyami MS . The prevalence of cardiovascular diseases, chronic kidney disease, and obesity in patients with type 2 diabetes mellitus and the description of concurrent treatments: A two-center retrospective cross-sectional study in Saudi Arabia. Saudi Pharm J. (2024) 32:102054. doi: 10.1016/j.jsps.2024.102054 38590611 PMC10999870

[31] Diabetes* ADAPPCf . 8. Obesity and weight management for the prevention and treatment of diabetes: Standards of care in diabetes–2026. Diabetes Care. (2025) 49:S166–82. doi: 10.2337/dc26-s008 41358882 PMC12690172

