# Supplementary material for: Comparative change in HbA1c and weight outcomes between obese and non-obese adults with type 2 diabetes mellitus treated with SGLT-2 inhibitors or GLP-1 receptor agonists
Source: Front Endocrinol (Lausanne). 2026 Aug 31;17:1847755. doi: 10.3389/fendo.2026.1847755 (PMC13572232; doi:10.3389/fendo.2026.1847755)
Supplement: Supplementary file 1 [file Supplementaryfile1.docx]

| **Table S1. Mean change in weight, BMI and HbA1c from baseline to follow-ups per medication group** | | | | | | |
| --- | --- | --- | --- | --- | --- | --- |
| **Characteristic** | **BMI < 30** | | | **BMI ≥ 30** | | |
|  | **GLP-1RA** | **SGLT-2i** | ***p*-value** | **GLP-1RA** | **SGLT-2i** | ***p*-value** |
| **Change in Weight, kg** |  |  |  |  |  |  |
| At 3 months follow-up | 0.16 ±3.9 | -0.49 ±3.8 | 0.3925 | -3.11 ±5.4 | -1.39 ±3.0 | **0.0002** |
| At 6 months follow-up | -1.46 ±5.0 | -0.88 ±3.4 | 0.5141 | -2.41 ±4.4 | -2.11 ±3.9 | 0.5590 |
| At 9 months follow-up | -1.98 ±5.4 | -0.36 ±3.1 | 0.1599 | -3.75 ±6.0 | -1.50 ±3.5 | **0.0003** |
| At 12 months follow-up | -0.20 ±4.7 | -0.76 ±3.2 | 0.5823 | -3.90 ±9.8 | -1.68 ±4.1 | **0.0315** |
| **Change in BMI, kg/m^2^** |  |  |  |  |  |  |
| At 3 months follow-up | 0.09 ±1.4 | -0.16 ±1.4 | 0.3673 | -1.20 ±2.1 | -0.55 ±1.1 | **0.0003** |
| At 6 months follow-up | -0.49 ±1.8 | -0.34 ±1.3 | 0.6551 | -0.92 ±1.7 | -0.84 ±1.6 | 0.6644 |
| At 9 months follow-up | -0.66 ±1.8 | -0.15 ±1.2 | 0.2088 | -1.46 ±2.3 | -0.64 ±1.4 | **0.0008** |
| At 12 months follow-up | -0.06 ±1.7 | -0.27 ±1.1 | 0.5801 | -1.44 ±4.0 | -0.67 ±1.6 | 0.0667 |
| **Change in HbA1c, %** |  |  |  |  |  |  |
| At 3 months follow-up | -0.95 ±1.5 | -0.70 ±1.8 | 0.4300 | -0.96 ±1.6 | -0.87 ±1.4 | 0.6025 |
| At 6 months follow-up | -1.07 ±1.8 | -0.89 ±1.6 | 0.5582 | -0.80 ±1.6 | -0.80 ±1.2 | 0.9770 |
| At 9 months follow-up | -0.77 ±1.5 | -0.99 ±1.8 | 0.5831 | -1.12 ±1.5 | -1.12 ±1.7 | 0.9737 |
| At 12 months follow-up | -0.60 ±1.8 | -0.54 ±1.5 | 0.8718 | -0.94 ±1.6 | -0.90 ±1.5 | 0.8620 |
| **Change in Fasting BG, mmol/L** |  |  |  |  |  |  |
| At 3 months follow-up | -1.64 ±2.6 | -0.94 ±4.6 | 0.4508 | -1.58 ±3.8 | -1.27 ±5.3 | 0.7089 |
| At 6 months follow-up | -0.45 ±3.3 | -1.81 ±5.0 | 0.2780 | -1.31 ±3.4 | -1.41 ±3.3 | 0.8461 |
| At 9 months follow-up | -1.73 ±2.7 | -0.31 ±6.5 | 0.2578 | -1.15 ±3.9 | -1.71 ±4.7 | 0.4620 |
| At 12 months follow-up | -1.68 ±3.4 | -1.25 ±3.2 | 0.6836 | -1.03 ±3.4 | -0.82 ±4.0 | 0.7813 |
| Numbers are presented as mean ±SD  Abbreviations: BMI: Body Mass Index; HbA1c: Hemoglobin A1c (glycated hemoglobin); BG: Blood Glucose. | | | | | | |

| **Table S2. Mean change in blood pressure and eGFR from baseline to follow-ups per medication group** | | | | | | |
| --- | --- | --- | --- | --- | --- | --- |
| **Characteristic** | **BMI < 30** | | | **BMI ≥ 30** | | |
|  | **GLP-1RA** | **SGLT-2i** | ***p*-value** | **GLP-1RA** | **SGLT-2i** | ***p*-value** |
| **Change in Systolic BP, mmHg** |  |  |  |  |  |  |
| At 3 months follow-up | -2.05 ±17.5 | -2.84 ±13.8 | 0.7859 | -2.65 ±16.3 | -1.76 ±16.8 | 0.6265 |
| At 6 months follow-up | -4.71 ±18.8 | -2.92 ±15.9 | 0.5799 | -1.41 ±14.9 | -4.04 ±16.2 | 0.1295 |
| At 9 months follow-up | -2.80 ±19.5 | -5.62 ±17.5 | 0.4937 | -3.61 ±18.2 | -3.10 ±17.3 | 0.8303 |
| At 12 months follow-up | 1.64 ±14.3 | -3.48 ±16.2 | 0.1606 | -3.35 ±14.9 | -2.11 ±17.0 | 0.5739 |
| **Change in Diastolic BP, mmHg** |  |  |  |  |  |  |
| At 3 months follow-up | -0.29 ±8.9 | -1.68 ±10.9 | 0.4793 | -0.08 ±9.8 | 1.56 ±12.1 | 0.1912 |
| At 6 months follow-up | -1.64 ±11.2 | -1.21 ±9.7 | 0.8265 | 0.47 ±10.0 | -0.40 ±11.9 | 0.4921 |
| At 9 months follow-up | 0.61 ±8.5 | -2.97 ±11.0 | 0.1295 | -0.83 ±11.4 | -0.17 ±12.4 | 0.6706 |
| At 12 months follow-up | 0.50 ±8.9 | -1.76 ±10.5 | 0.3355 | -1.26 ±9.9 | 0.04 ±10.7 | 0.3636 |
| **Change in eGFR, ml/min/1.73m^2^** |  |  |  |  |  |  |
| At 3 months follow-up | 2.11 ±12.5 | -1.19 ±15.1 | 0.2794 | -0.42 ±16.6 | -1.52 ±15.7 | 0.5781 |
| At 6 months follow-up | 0.00 ±10.8 | 1.54 ±12.3 | 0.5263 | -1.12 ±14.6 | -1.34 ±16.3 | 0.9085 |
| At 9 months follow-up | 0.09 ±15.1 | 1.92 ±13.2 | 0.5968 | -2.59 ±15.4 | -0.04 ±15.3 | 0.2101 |
| At 12 months follow-up | -0.72 ±12.3 | -1.58 ±13.3 | 0.7919 | -1.35 ±16.4 | -4.36 ±17.1 | 0.2194 |
| Numbers are presented as mean ±SD  Abbreviations: BP: Blood Pressure; eGFR: estimated Glomerular Filtration Rate. | | | | | | |

| **Table S3. Mean change in weight and HbA1c from baseline to follow-ups within each medication class** | | | | | | |
| --- | --- | --- | --- | --- | --- | --- |
| **Characteristic** | **GLP-1RA** | | | **SGLT-2i** | | |
|  | **BMI < 30** | **BMI ≥ 30** | ***p*-value** | **BMI < 30** | **BMI ≥ 30** | ***p*-value** |
| **Change in Weight, kg** |  |  |  |  |  |  |
| At 3 months follow-up | 0.16 ±3.9 | -3.11 ±5.4 | **<0.0001** | -0.49 ±3.8 | -1.39 ±3.0 | 0.0950 |
| At 6 months follow-up | -1.46 ±5.0 | -2.41 ±4.4 | 0.2304 | -0.88 ±3.4 | -2.11 ±3.9 | **0.0263** |
| At 9 months follow-up | -1.98 ±5.4 | -3.75 ±6.0 | 0.1593 | -0.36 ±3.1 | -1.50 ±3.5 | **0.0472** |
| At 12 months follow-up | -0.20 ±4.7 | -3.90 ±9.8 | **0.0056** | -0.76 ±3.1 | -1.68 ±4.1 | 0.1792 |
| **Change in BMI, kg/m^2^** |  |  |  |  |  |  |
| At 3 months follow-up | 0.09 ±1.4 | -1.20 ±2.1 | **<0.0001** | -0.15 ±1.4 | -0.55 ±1.1 | **0.0403** |
| At 6 months follow-up | -0.49 ±1.8 | -0.92 ±1.7 | 0.1418 | -0.34 ±1.3 | -0.84 ±1.6 | **0.0248** |
| At 9 months follow-up | -0.66 ±1.8 | -1.46 ±2.2 | 0.0911 | -0.15 ±1.2 | -0.64 ±1.4 | **0.0393** |
| At 12 months follow-up | -0.06 ±1.7 | -1.44 ±4.0 | **0.0069** | -0.27 ±1.1 | -0.67 ±1.6 | 0.1036 |
| **Change in HbA1c, %** |  |  |  |  |  |  |
| At 3 months follow-up | -0.95 ±1.5 | -0.96 ±1.6 | 0.9641 | -0.70 ±1.8 | -0.87 ±1.4 | 0.4513 |
| At 6 months follow-up | -1.07 ±1.8 | -0.80 ±1.6 | 0.3215 | -0.89 ±1.6 | -0.80 ±1.2 | 0.6910 |
| At 9 months follow-up | -0.77 ±1.5 | -1.12 ±1.5 | 0.2510 | -0.99 ±1.8 | -1.12 ±1.7 | 0.6432 |
| At 12 months follow-up | -0.60 ±1.8 | -0.94 ±1.6 | 0.3045 | -0.54 ±1.5 | -0.90 ±1.5 | 0.1799 |
| Numbers are presented as mean ±SD  Abbreviations: HbA1c: Hemoglobin A1c (glycated hemoglobin). | | | | | | |
